# The association between chronic alcohol, cannabis, and opioids use and autobiographical memory impairments: a systematic review

**DOI:** 10.3389/fpsyt.2026.1715085

**Published:** 2026-02-19

**Authors:** Molly Gibson, Zeynab Hemmati, Aldo Conti, Barbara Dritschel, Alexander Baldacchino

**Affiliations:** 1 School of Medicine, The University of Manchester, Manchester, United Kingdom; 2 Department of Radiology, Salford Royal Hospital, Northern Care Alliance, NHS Foundation Trust, Salford, United Kingdom; 3 Department of Child and Adolescent Psychiatry, Institute of Psychiatry, Psychology and Neuroscience, King’s College London, London, United Kingdom; 4 Florence Nightingale Faculty of Nursing, Midwifery and Palliative Care, Division of Care in Long Term Conditions, King’s College London, London, United Kingdom; 5 School and Psychology and Neuroscience, University of St Andrews, St Andrews, United Kingdom; 6 Division of Population and Behavioural Sciences, University of St Andrews School of Medicine, St Andrews, United Kingdom

**Keywords:** alcohol, autobiographical memory, cannabis, chronic, opioids, psychoactive substance

## Abstract

**Introduction:**

Evidence suggests an association between chronic psychoactive substance use and memory deterioration. Autobiographical memory (AM) is one form of long-term memory that is captured through specific personal information. Exploring potential causes of AM impairment is crucial as these memories shape identity and are important for problem solving and imagining the future. This is the first systematic review to primarily assess the association between chronic psychoactive substance use, including cannabis, alcohol, and opioids, and AM impairment. Additionally, associations between AM performance and secondary outcome measures encompassing mental health, severity and pattern of substance use, cognitive and emotional functions, Theory of Mind and Fading Affect Bias were also explored in this paper.

**Methods:**

.An extensive literature search was conducted using PRISMA guidelines for systematic reviews and meta-analyses. The review covered articles from 2003 to 2025 obtained from searching 10 global databases. Relevant articles were then screened for eligibility based on the inclusion and exclusion criteria for the systematic review. They were also screened for bias and quality. This resulted in 13 papers being narratively synthesized.

**Results:**

The narrative synthesis showed a strong association between the chronic use of different substances (e.g. alcohol, cannabis, opioids) and AM impairments. Those chronically using alcohol, cannabis, and opioids consistently retrieved less specific and more general AMs compared to controls. It could also be postulated that AM impairments may be related to executive dysfunctions caused by the daily consumption of psychoactive substances. However, direct causality cannot be inferred due to the cross-sectional design of the studies pooled for the current review.

**Summary:**

Future research needs to expand further the association between chronic psychoactive substance use and AM impairments as this will be clinically relevant for treatment planning in this population.

## Introduction

Substance use disorder is a “cluster of cognitive, behavioural, and physiological symptoms indicating that the individual continues using the substance despite significant substance related problems”. The Diagnostic and Statistical Manual of Mental Disorders, 5^th^ Edition (DSM-5-TR) outlines 11 diagnostic criteria that can be used to assess the severity as mild, moderate, or severe substance use disorder (1). Chronic substance use refers to a period of 12 months or more; a diagnosis can also be made if use is daily or almost daily for 3 months or more (International Classification of Diseases, 11^th^ Revision) (2). According to the World Drug Report 2024, an estimated 64 million people worldwide were suffering from a drug use disorder in 2022, a 3% increase from 2018 with the most consumed drug globally remaining as cannabis, followed by opioids (3). The United Nations Alcohol Report 2024 estimated that 7% of the global population are living with alcohol use disorders (4). Scientific literature has proposed a strong association between the chronic use of a wide range of psychoactive substances and impairments in several neuropsychological domains (5–9), including long-term memory, short-term memory, and working memory impairments (6–8). Recent research has also proposed that substance use may negatively impact Autobiographical Memory (AM) (10–12). AM can be divided into episodic AM, which refers to the memory for personally experienced events set in a spatiotemporal context, and semantic AM, which refers to factual knowledge about the individual (13, 14). AM forms the foundation of understanding oneself, and guides problem solving and future behaviours, in turn allowing social connections to be established and maintained (15). It also plays an important role in emotion regulation (16). According to the Self Memory System framework (17), AM is organised hierarchically. At the top of the hierarchy is knowledge about lifetime periods (e.g., my time at university). At the intermediate level there are general memories which consist of repeated categoric events (e.g., going to statistics lectures on Thursdays) as well as extended events (e.g., a ski trip in France with the skiing society). Finally, at the bottom of the hierarchy, there are specific memories which are memories for highly contextualised events in terms of time and place (e.g., receiving my acceptance letter for university). As specific memories are at the bottom of the hierarchy their retrieval is more cognitively demanding (17). Difficulties in retrieving specific memories are a transdiagnostic feature of multiple conditions including depression (18), post-traumatic stress disorder (19), schizophrenia (20) and eating disorders (21). According to the Carfax model (22), several mechanisms can explain the difficulty in retrieving specific memories. They include rumination, the tendency to think perseveratively about the causes and consequences of one’s mood, functional avoidance, and executive functioning difficulties. Rumination is an abstract negative thinking style that promotes overgeneral retrieval as it disrupts retrieval processes by making it difficult to shift thinking beyond abstract, negative, self-schematic thinking. Overgeneral memories are more abstract than specific memories, increasing the likelihood that memory retrieval will be captured at this stage. According to the functional avoidance account, overgeneral recall occurs because overgeneral memories contain less contextual detail than specific memories and therefore are less likely to reactivate emotional experience associated with the event (23). Finally, overgeneral memory requires fewer executive resources to retrieve than specific memory recall. Reduced executive function can lead to difficulties with refinement of the retrieval process (24).

One feature associated with AM, that may be important for understanding the impact of substance use on AM, is the emotional valence of the memory which can be described as positive, negative, or neutral (25). The effects of emotional valence on AMs are explained by two cognitive phenomena occurring at separate points in the memory encoding and retrieval process. Firstly, The Mood Congruent Effect (MCE) suggests that affect experienced during an event impacts the valence associated with its memory (26). For example, if an individual experiences emotional distress during an event, the memory will be associated with a negative valence, even if the scenario reflects a typically positive experience (e.g., attending a party), suggesting that mood serves as a cue for memory retrieval. Secondly, the Mood Dependent Effect (MDE) implies that affect experienced at the point of memory retrieval influences how the memory is recalled, making it vulnerable to inaccurate representation (27). Considering that individuals affected by substance use disorders display poor emotional regulation (28), and that drug withdrawal symptoms usually include negative mood states (e.g. depression, anxiety, irritability) (29, 30), those chronically using substances may be particularly prone to retrieve negatively skewed AMs. These AM impairments may hamper the ability of individuals exhibiting chronic substance use disorder to construct an accurate sense of self and identity that reflects positive attributes (31).

AM retrieval involves a large neural network including the Prefrontal Cortex (PFC), hippocampus, parahippocampal cortex, cingulate gyrus, angular gyrus and the limbic system (e.g., amygdala). Each region of this network is activated during a different cognitive process pertaining to AM retrieval (32). Despite the substantial body of literature showing an association between chronic substance use and neurofunctional/neuroanatomical impairments in the aforementioned brain areas (33–38), there is still a lack of empirical data investigating the association between chronic substance use and AM impairments. Wright et al., 2022 (39) conducted a systematic review to investigate Self- Defining Memories (SDM) characteristics among individuals with substance use, mental, cognitive, neurodevelopmental, and physical health impairments. Self-defining memories represent key aspects of one’s identity (40). Cousijn et al., 2012 (41) reported that individuals affected by Opioid Use Disorder and Alcohol Use Disorder (AUD) recalled fewer specific AMs compared to controls. However, their findings were limited to the qualitative summary of three studies as the authors only pooled studies that used the Self-Defining Memories Questionnaire (SDMQ) (40) as an outcome measure. According to Wright et al., 2022 (39), the use of a single measure of AM is an important limitation as studies that assessed AMs using alternative methods may have been missed from the narrative synthesis. Such methods include the autobiographical memory test (AMT) (42), where AMs (typically specific memories) are retrieved to cues under timed conditions, the Sentence Completion for Past Events Test (SCEPT) (43), where participants are asked to complete sentence fragments by retrieving personal memories, and the Autobiographical Memory Interview (AMI) (44), where episodic and semantic information from different lifetime periods are recalled in response to structured questions.

To understand the impact of substance use on AM, associations between AM performance and secondary outcome measures encompassing mental health states, severity and pattern of substance dependence, Theory of Mind, and Fading Affect Bias have also been explored. Theory of Mind was first proposed by Premack and Woodruff et al., 1978 (45) as an integral component of social interactions and understanding, as it is the ability to comprehend and consider the mental states of those around you. Therefore, impaired abilities to recognise emotions can influence the encoding and retrieval of AMs according to the Mood Dependent and Mood Congruent Effects (26). Additionally, Fading Affect Bias has the ability to influence the retention of AMs, with memories of negative valence fading at a faster rate than those of positive valence (46). If the fading affect bias is impaired in substance use, negative mood may be maintained.

Considering the above gap in literature and the need to assess the influence of secondary measures further, this systematic review aims to elucidate and summarise:

1. The relationship between chronic psychoactive substance use disorder and different facets of AM impairment.
2. The association between secondary outcome measures (e.g., depression, anxiety, severity, pattern of substance use, theory of mind and the fading affect bias) and AM performance.

## Methods

### Study identification and selection

**Figure 1:**
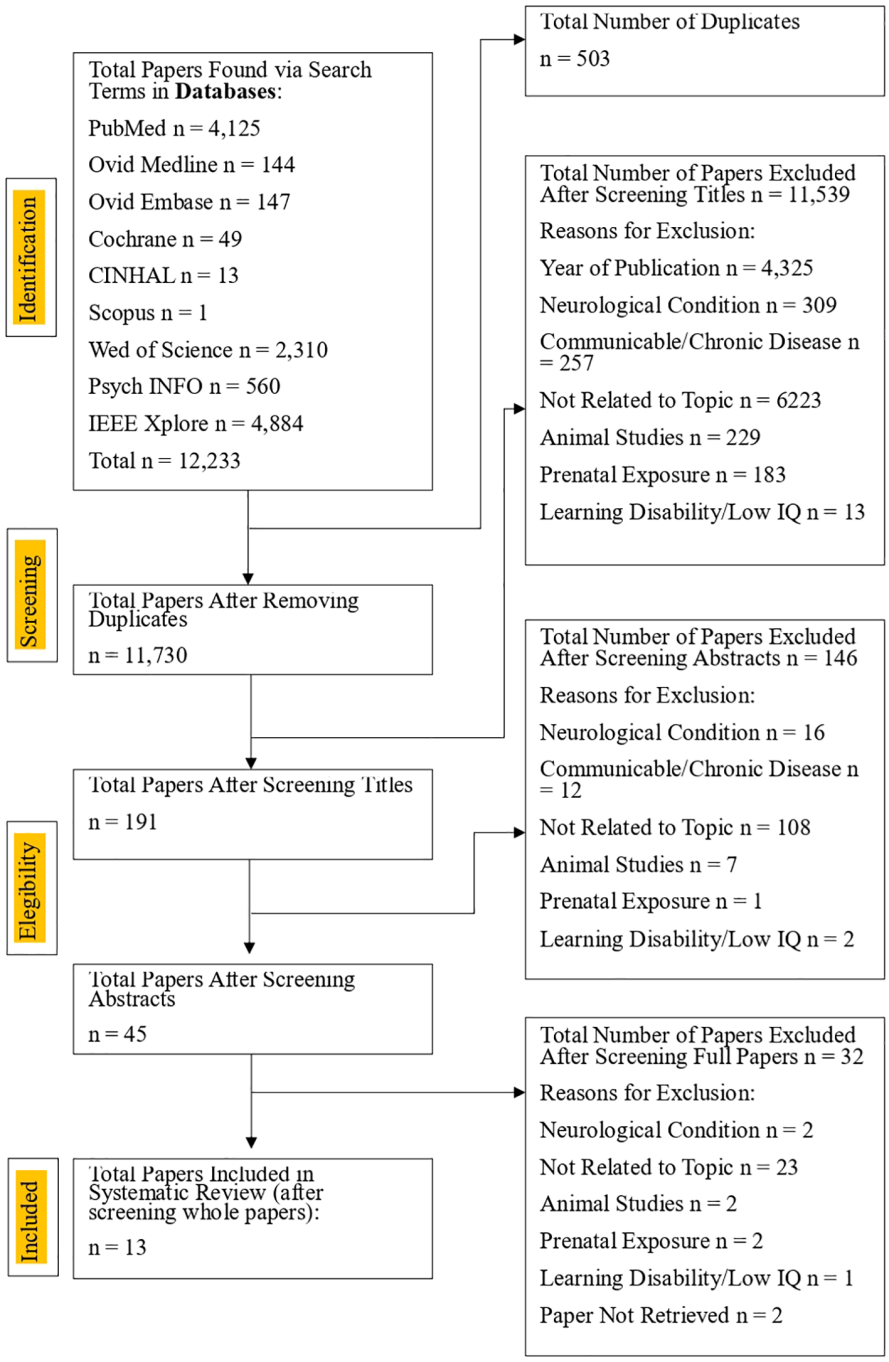
A PRISMA chart showing the screening process used to select papers to be included in the review.

This systematic review was conducted according to the preferred reporting items for systematic reviews and meta-analysis (PRISMA) guidelines (47). The following databases were searched until September 22^nd^ 2025: PubMed (via NCBI), Cochrane Central Register of Controlled Trials (CENTRAL), CINAHL (via EBSCOhost), PsychINFO (via ProQuest), Scopus, Web of Science (including Biosis, Scielo and KCI-Korean Journal), Medline (via OVID), Embase (via OVID) and IEEE Xplore Digital Library. The PICO was established to determine the search terms: male and females who use chronic psychoactive substances (population), possible effects of chronic psychoactive substance use on AM (intervention), in comparison to controls (control), and assessment of several aspects of AM functioning in those chronically using psychoactive substances (outcome). Subcategories of the search terms were then implemented: AM, psychoactive substances, chronic use, impairment, and polysubstance use. The extensive list of search terms entered into each database can be found in **Table 1**. Identified papers were first manually screened by title and abstracts for duplicates and checked for relevance, then analysed fully in line with the eligibility criteria. The PRISMA chart depicted in **Figure 1** outlines the identification and selection process.

**Table 1:** Database search terms.

Subcategories	Search terms
Autobiographical Memory	Autobiographical memory OR personal episodic memory OR personal semantic memory OR personal memory OR autobiographical episodic memory OR autobiographical semantic memory OR self-centred memory OR autobiographical memory specificity OR general autobiographical memory OR childhood memory OR life events OR narrative memory OR positive life events OR negative life events OR central life events OR voluntary autobiographical memory OR involuntary autobiographical memory OR memory for future events OR trauma memories AND
Psychoactive Substances	Analgesics OR opioid* OR opiate alkaloids OR opioid peptides OR opium OR opiate substitution treatment OR fentanyl OR papaverine OR noscapine OR naloxone OR naltrexone OR buprenorphine OR nalorphine OR nalbuphine OR morphine OR codeine OR hydrocodone OR oxycodone OR dihydromorphine OR heroin OR hydromorphone OR oxymorphone OR etorphine OR diprenorphine OR dextromethorphan OR pethidine OR piritramide OR remifentanil OR tramadol OR oramorph OR oxycontin OR alfentanil OR desomorphine OR endomorphin OR enkephalin OR lofentanil OR methadone OR normethadone OR sufentanil morphine OR opiate* OR amphetamine type stimulant* OR amphetamine* OR methamphetamine* OR dexamphetamine* OR methylamphetamine OR dextroamphetamine OR dexedrein OR nicotine* OR smok* OR tobacco* OR pipe OR cigar OR passive smoking OR snuff OR betel nut OR shisha OR alcohol* OR ethanol* OR alcohol abuse OR alcohol consumption OR cannabis OR cannabinoids OR marijuana OR joint OR THC OR tetrahydrocannabinol OR weed OR benzodiazepine* OR diazepam OR flurazepam OR clobazam OR halazepam OR loprazelam OR cloxazelam OR midazolam OR oxazolam OR triazolam OR lorazepam OR quazepam AND
Chronic Use	Chronic OR abuse* OR addict* OR misuse OR long-term OR disorder OR depend* OR excessive use OR repeated use OR overuse AND
Impairment	Test* OR deficit OR impairment OR cognitive decline OR amnesia OR memory loss AND
Polysubstance Use	Polydrug* OR polysubstance* OR substance use OR substance use disorder OR drug use disorder OR drug abuse OR substance abuse OR polydrug abuse

### Eligibility criteria

The inclusion criteria were the following: randomised control trials, case reports, case series, research reports and other grey literature in line with the review’s PICO. The exclusion criteria were the following: a lack of chronic psychoactive substance use, no AM cognitive testing, self-reporting, prenatal studies, animal studies, systematic reviews and meta-analyses, studies including participants with severe learning disabilities, low IQ, or chronic or communicable diseases and neurological conditions including Korsakoff’s syndrome. To further refine the search, the years of publication were limited to January 2003 – September 2025, and the language to English, Spanish, French, or Italian. No age limits were set.

### Bias and quality assessment

Publication bias refers to the tendency to publish studies reporting statistically significant results instead of studies reporting results that are not statistically significant (48, 49). Therefore, there is a possibility that studies included in a systematic review would be biased and consequently reflected in the results of the narrative synthesis (7). The Joanna Briggs Institute Checklist (50) for analytical cross-sectional studies was utilised to assess bias and quality across the included studies, the results are outlined in **Table 2**.

**Table 2:** A Quality assessment of the literature using JBI (50).

Paper	Inclusion criteria clearly stated	Participant demographics and setting clearly described	Exposure to substance reliably measured	Standard criteria to measure AM function	Confounding factors identified	Confounding factors addressed	Adequate measure of outcomes	Adequate statistical analysis	Paper included in review?
D’Argembeau et al., 2006 (51)	YES	YES	YES	YES	YES	YES	UNCLEAR	YES	YES
Poncin et al., 2015 (52)	YES	YES	YES	YES	YES	NO	UNCLEAR	YES	YES
Pillersdorf et al., 2018 (53)	YES	YES	YES	YES	YES	YES	YES	YES	YES
Nandrino et al., 2014 (54)	YES	YES	YES	YES	YES	YES	YES	YES	YES
Nandrino et al., 2016 (55)	YES	YES	YES	YES	YES	YES	YES	YES	YES
Nandrino et al., 2017 (31)	YES	YES	YES	YES	YES	YES	YES	YES	YES
Gandolphe et al., 2011 (56)	YES	YES	UNCLEAR	UNCLEAR	YES	YES	UNCLEAR	YES	YES
Gandolphe et al., 2013b (57)	YES	YES	YES	UNCLEAR	YES	YES	UNCLEAR	YES	YES
Gandolphe et al., 2013a (58)	YES	YES	YES	UNCLEAR	YES	YES	YES	YES	YES
Gandolphe et al., 2019 (59)	YES	YES	YES	YES	YES	YES	UNCLEAR	YES	YES
Cuervo- Lombard et al., 2016 (60)	YES	YES	YES	YES	YES	YES	UNCLEAR	YES	YES
De Groote et al., 2023 (61)	YES	YES	YES	YES	YES	YES	UNCLEAR	YES	YES
De Groote et al., 2025 (62)	YES	YES	YES	YES	YES	YES	UNCLEAR	YES	YES

### Narrative synthesis

A narrative synthesis methodology (63) was used to summarise and describe the results of studies investigating the effects of chronic psychoactive substance use on AM. It was not possible to conduct a meta-analysis due to data unavailability.

## Results

The database search produced a total of 12,233 papers, which were screened for duplicates and relevance, resulting in 45 papers. The papers were then checked in line with the eligibility criteria and PICO, which led to the inclusion of 13 papers in this review. The selection process is outlined in **Figure 1**.

### Characteristics of the included studies

The 13 papers were conducted between 2003 and 2025 in France (31, 52, 54–59, 61, 62)), Switzerland (60), Belgium (51), and Canada (53). The studies were selected following a systematic search of global databases to help ensure that outcomes are generalisable to the widest population possible. The age of participants across the studies ranged from 21 years to 57 years. Collectively, the control group consisted of 441 participants and the cohort of 578 participants who use substances. Further demographic variables are outlined in **Table 3**. Across the 13 papers, 3 psychoactive substances were primarily explored, including alcohol ( (31, 51, 52, 54, 55, 60–62), cannabis (53, 56) and opioids (57–59). Polysubstance use was explored in a proportion of these papers. Cuervo-Lombard et al., 2016 (60) was the only study to investigate alcohol use both alone and in the context of polysubstance use with benzodiazepines or nicotine. Gandolphe et al., 2011 (56) explored cannabis use with subsequent alcohol exposure whilst Pillersdorf et al., 2018 (53) isolated cannabis use alongside unspecified polysubstance use disorder, in addition to the controls. Finally, Gandolphe et al., 2019 (59) investigated isolated opioid use disorder compared to opioid use in addition to cannabis, cocaine, alcohol, or benzodiazepine use. All remaining studies investigated single substance use disorder. Tobacco was not excluded by Cuervo-Lombard et al., 2016 (60), Poncin et al., 2015 (52) or Nandrino et al., 2016 (55). Likewise, nicotine and anti-craving medications were not excluded by De Groote et al., 2023 (61) or De Groote et al., 2025 (62), but this was not considered polysubstance use disorder.

**Table 3:** Study characteristics and results.

Author and location	Participant groups and demographics		Length of consumption	Condition at the time of testing	AM type	AM outcome measure	Reported results	Summary of findings
	Those using substances	Controls						
Alcohol								
De Groote et al., 2025 (62) France	N=37 M(62)=49.7 &.7) yrs 41% Female	N=37 M(62)=49.5 (9.8) yrs 41% Female	M(62)=16.2 (10.6) yrs	Abstinence (N = 37; M(62)=26.8 (38.2) days)	Subjective experiences of memories	MEQ-SF (Luchetti et al., 2016) (64)	Increased distancing from AMs was observed in the individuals who had an alcohol use disorder, with levels varying at different stages of life ((young adulthood p=0.04); adulthood p<0.001; past year p<0.001). Negative valence of memories was also significantly higher in those with alcohol use disorder (adulthood p=0.023); past year p=0.009).	“The results showed several phenomenological differences for AMs from the last year, whereas few phenomological intergroup differences were observed for AMs from childhood, adolescence and young adulthood, and adulthood. In addition, the alcohol use dependent group experienced greater feelings of distancing than did the control group for all the AMs expect from those from childhood, indicating a poor diachronic unity of self.”
Cuervo-Lombard et al., 2016 (60) Switzerland	N=25 M(62)=44.6 (7.0) yrs 32% Female	N=28 M(62)=41.9 (8.7) yrs 42% Female	M(62)=30 (8.0) yrs	Abstinence (N = 25; M(62)=23 (31.7) days)	Self-defining memories	SDQ (singer and Moffitt 1991 (40))	Those using alcohol reported significantly fewer specific SDMs than control (p=0.016). SDMs had significantly more negative valence in participants using alcohol (p=0.006) and the intensity of negative emotional responses was significantly higher (p=0.003) compared to controls.	“Our main finding was that SDMs in alcohol dependent individuals were significantly less specific and contained more reference to alcohol than those of controls. Alcohol dependent individuals reported more SDMs with negative emotional valence than controls and with higher emotional intensity. SDMs can be used as a predictor of relapse and for specific psychotherapy.
D’Argembeau et al., 2006 (51) Belgium	N=20 M(62)=44.7 (8.4) yrs 15% Female	N=20 M(62)=44.6 (8.8) yrs 15% Female	n/a	Abstinence (N = 25; M(62)=19.5 (2.6) days	Specific Recall	AMT (Williams and Broadbent 1986) (42)	The frequency of specific memories was significantly lower in those using alcohols than in controls (p=0.001). Both extended and categoric memories were significantly more frequent in people consuming alcohol than in controls (p<0.05).	“The results of the AMT showed that alcoholics reported specific memories less frequently and categoric extended memories more frequently than healthy controls … the reduced ability of alcohol dependent individuals to retrieve specific memories might place them in a less comfortable position when dealing with current challenges in their social environment, and this might be a risk factor for having a relapse…”
Nandrino et al., 2014 (54) France	N=50 M(62)=45 (8.71) yrs 16% Female	N=30 M(62)=43.8 (10.7) yrs 10% Female	M≥1 year	Abstinence (N = 50; M≥3 weeks)	Episodic ad Semantic AM	AMI (65) (Kopelman et al., 1994)	Alcohol dependent subjects had significantly lower scores than controls for both episodic (p=0.0001) and semantic (p=0.01) autobiographical memory.	“In alcohol dependent individuals, our results show that they have AM difficulties at both semantic and episodic levels … This lack of structuring of autobiographical memories (temporal and semantic organisation) could explain the increased sensitivity to social event and difficulty in adapting to social interaction for AD subjects…”
Poncin et al., 2015 (52) France	N=61 Short-term abstinence: N=41 M(62)=51.2 (11.02) yrs 32% Female Lon-term abstinence: N=20 M(62)=59.7 (10.4) yrs 35% Female	N=20 M(62)=51.1 (12.3) yrs 40% Female	Short-term abstinence: M(62)=14.5 (11.28) yrs Long-term abstinence: M(62)=12.8 (9.14) yrs	Short-term abstinence: N=41; M(62)=15 (0) days Long-term abstinence: N=20 M(62)=129.1 (129.9) months	Specific recall	AMT (Williams and Broadbent 1989) (42)	Participants using alcohol (short-term abstinence) provided a significantly lower proportion of specific memories than controls and long-term abstinence subjects (p<0.01).	“Alcohol-dependent subjects presented with reduced access to specific autobiographical memories. The access the autobiographical memory specificity was better in alcohol-dependent subjects who had all been abstinent for more than 6 months, than in alcohol-dependent subjects (short-term abstinence) and equivalent to that of healthy control subjects. These observations support the possibility of recovery of episodic memory capacities with abstinence…”
Nandrino et al., 2016 (55) France	N=57 Short-term abstinence: N=n/a M(62)=42.7 (8.5) yrs Long-term abstinence: N=n/a M(62)=45.1 (7.3) yrs 23% Female	N=35 M(62)=45.9 (7.5) yrs 40% Female	M≥1 year	Short-term abstinence: N=n/a M≥3 wks Long-term abstinence: N=n/a M≥6 mths	Episodic and semantic AM	AMI (65) (Kopelman et al., 1994), SRT (66) (Randall et al., 2011)	The short-term abstinence group had significantly lower mean scores for episodic (p<0.0001) and semantic (p=0.035) AM than the control group. The long-term abstinence group had significantly lower mean scores for episodic (p<0.0001) and semantic (p=0.013) AM than the control group.	“Diminished semantic and episodic AM performance was observed across the life periods for both the semantic and episodic components … regardless of the abstinence duration, both patient groups showed the same pattern of AM problems. This suggests that impairment sin episodic and semantic memory persists throughout these abstinence periods … AM impairments appear to be stable over time or may need a longer recovery period…”
Nandrino et al., 2017 (31) France	N=27 M(62)=50.4(7.0) 30% Female	N=28 M(62)=n/a 46% Female	M(62)=11.5 (10.83) yrs	Abstinence: N=27 M≥2mths	Self-defining memories	SDQ (singer and Moffitt 1991) (40)	A significantly lower frequency of specific SDM (p<0.01) and a lower frequency of integrated SDM recall (p=0.001) was found in those using alcohol compared to controls. A significantly lower frequency of positive SDM (p<0.001) and a significantly higher frequency of negative (p<0.01) and neutral (p<0.01) SDM was found in people using alcohol compared to controls. Controls recalled the themes of “achievement” and “mastery” with a significantly higher frequency compared to the group using alcohol (p<0.01).	“Overall, when we compared the SDM of the 2 groups, we found that they were specifically characterised by: (i) low specificity, (ii) low integration, (iii) a predominance of memories with negative emotional valence and a low frequency of positive memories, and (iv) a low frequency of topics related to success … these results point to the construction of a specific form of SDM with drinking problems mainly characterised by the disruption of positive memories and the presence of highly specific and integrated negative experiences … such a disruption could constitute one of the main risks if continued drinking problems and could stop the individual from exiting the vicious circle of repeated negative memories.”
De Groote et al., 2023 (61) France	N=31 M(62)=44.7 (10.7) yrs 45% Female	N=32 M(62)=44.7 (10.2) yrs 28% Female	M(62)=20.3 (8.8) yrs	Abstinence: N=31 M(62)=63.3 (112.6) weeks	Specific recall, AM valence	IAM Task (Rathbone et al., 2008) (67)	A significant association was reported between the ‘group’ variable and number of negative self-statements (p=0.015), and between the ‘group’ variable and number of neutral self0statements (p=0.024). A significant association was reported between the ‘group’ variable and specificity levels (p<0.001), and between the ‘group’ variable and number of general memories (p<0.001). A significant association was reported between the ‘group’ variable and number of positive memories (p<0.001), and between the ‘group’ variable and the recalled “achievement”, “alcohol”, and “recreation” themes (p<0.05).	“Our results showed that the group was related to both negative and neutral self-statements, such the alcohol use disorder individuals reported more negative self-statements and fewer neutral self-statements than would have been expected without these relations … it is possible that negative self-descriptive words are more accessible and neutral self-descriptive words less accessible in alcohol use disorder patients rather than in control participants’ semantic autobiographical knowledge … moreover … individuals retrieved a higher number of general memories … general autobiographical memories might prevent alcohol use disorder individuals from having a rich, contextualised and nuanced self-understanding … individuals recalled fewer positive memories … this lower ability to recall positive autobiographical memories may result from an impaired encoding of past events associated with positive emotions or from a difficulty to accept positive information in alcohol use disorder individuals … alcohol use disorder individuals recalled fewer memories about achievements tan expected. Thet might define themselves less based on their personal achievements…
Cannabis								
Pillersdorf et al., 2018 (53) Canada	N=47 M962)=21.2 (2.71) yrs 71% Female	N=52 M(62)=21.2 (2.71) 71% Female	M≥1 year	Active consumption – minimum 3x per month over the past year (N = 47; M(62)=n/a)	Specific recall and fading affect bias	SCEPT (43) (Raes and Hermans et al., 2007), Fading affect bias protocol (Cason et al., 1932 (68))	Those using cannabis provided a significantly higher proportion of over general memory responses (OGM) on average compared to those who do not use cannabis (p<0.05). The affect associated with memories for unpleasant events decreased significantly (p<0.05) more in those not using cannabis compared to those that do.	“The results suggested that cannabis disrupts the emotional regulation of affect tied to unpleasant autobiographical events and it disrupts memory specificity in that it is connected to over general memory … documenting the effects of cannabis is important given the global trend toward the legalisation of cannabis for recreational purposes…”
Gandolphe et al., 2011 (56) France	N=69 People occasionally to regularly using cannabis: N=17 M(62)=21.8 (2.7) years 6% Female People regularly using cannabis: N=17 M(62)=22.0 (3.0) yrs 6% Female People dependent on cannabis: N=17 M(62)=21.2 (3.1) yrs 12% Female People using cannabis with other substances: N=18 M(62)=25.6 (4.5) yrs 17% Female	N=38 M(62)=22.8 (3.7) 13% Female	n/a	Active consumption People who occasionally to regularly use cannabis: N=17 M(62)=18.5 (14.3) times per month People who regularly use cannabis: N=17 M(62)=154.5 (94.4) times per month People depended on cannabis: N=17 M(62)=362.9 (121.3) times per month	Specific Recall	AMT (Williams and Scott 1988)	Individuals dependent on cannabis and those who use multiple substances recalled a significantly higher percentage (p<0.001) of general memories compared to those regularly using cannabis regularly, occasionally – regularly and not at all. No statistically significant difference was noted between individuals dependent on cannabis and those who use multiple substances (p=1.00) and nor between those using cannabis occasionally to regularly (p=0.13). No statistically significant difference was also reported between controls and those occasionally using cannabis (p=0.6).	“…Individuals with cannabis use disorder and individuals dependent on a substance are more likely to favour a general mode of recall than control individuals … The results … could suggest that this phenomenon is related to memory disturbances induced by substance use … the retrieval of autobiographical memories requires the use of executive functions, the alteration of which generates a difficulty in accessing specific memories … autobiographical memory disturbances can also be thought of as a functional avoidance strategy…”
Opioids								
Gandolphe et al., 2013b (57) France	N=73 Active consumption: N=30 M(62)=27.4 (6.1) years 13% Female Methadone maintenance: N=23 M(62)=33.5 (6.9) years 22% Female Partial Remission: N=20 M(62)=30.5 (4.3) years 15% Female	N=37 M962)=25.4 (9.4) 19% Female	Active consumption: N=30 M(62)=7.9 (4.2) years Methadone maintenance previous opioid use: M(62)=9.4 (4.9) years Partial remission previous opioid use: M(62)=9.45 (3.8) years	Active consumption: N=30 Methadone maintenance: N=23 M(62)=51.1 (44.3) months Partial remission: N=20 M(62)=25.7 (37.0) months	Specific recall	AMT (Williams and Scott 1988)	The active consumption, methadone maintenance, and partial remission groups combined recalled a significantly lower percentage of specific memories compared to controls (p<0.001). No statistically significant differences were reported between the active consumption, methadone maintenance and partial remission groups regarding the percentage of specific memories recalled (p>0.05).	“The autobiographical memory test results show that multiple substance-dependent individuals have reduced autobiographical memory specificity compared to control individuals … The results show that neither methadone treatment nor withdrawal have an immediate impact on … memory specificity … These findings are consistent with the literature asserting that cognitive impairments persist after a withdrawal period … It is already known that autobiographical memory retrieval requires executive control, the impairment of which would lead to difficulty in accessing specific memories”
Gandolphe et al., 2013a (58) France	N=30 M(62)=28.6 (6.4) years 27% Female	N=30 M(62)=29.5 (4.9) 27% Female	M(62)=10.1 (6.2) years	Early abstinence: N=30 M(62)=5.0 (3.7) days	Specific recall	AMT (Williams and Scott 1988)	Those using opioids recalled a significantly smaller proportion of specific memories compared to controls (p<0.01).	“The autobiographical memory test results confirm the prevalence of a more general memory retrieval mode in opioid-dependent individuals … reduced specificity can be considered an emotional avoidance strategy … reduced specificity has been recognised as being associated with difficulty in imagining future events with less effective problem solving…”
Gandolphe et al., 2019 (59) France	N=25 M(62)=36,3 (7.1) 28% Female	N=25 M(62)=36.7 (9.9) 32% Female	M(62)=10.4 (6.9) years	Abstinence: N=25 M(62)=55.3 (93.1) days	Self-defining memories	SDQ (Singer and Blagov 2002) Reference	Those using opioids had a significantly lower probability of recalling specific SDMs compared to control (p<0.01). People using opioids had a significantly lower probability of recalling integrated SDM compared to controls (p<0.05). The group of people using opioids had a significantly higher probability of recalling a neutral memory compared to controls (p<0.05). No statistically significant differences were reported regarding the probability of recalling negative and positive events and themes between people using opioids and controls (p>0.05).	“The comparison of SDM in opioid-dependent individuals with those of non-dependent individuals showed that the SDM in the clinical population were less specific, had more neutral content and were less integrated. However, the themes mentioned by the opioid-dependent individuals in their SDM, as well as the emotions triggered during the SDM retrieval in terms of valence and arousal, were not statistically significant when compared to those of the non-dependent individuals … These results seem to reflect a global impoverishment of past experiences in both the emotional and cognitive aspects that opioid-dependent individuals may be unaware of and may constitute a major risk for relapse as it impedes personal adjustment…”

### Chronic alcohol use and autobiographical memory

The 8 papers exploring alcohol as the primary substance reported impairment in one or more functions of AM in those chronically using alcohol, compared to controls. Some of the studies refer to the group using alcohol as alcohol-dependent (AD). Cuervo-Lombard et al., 2016 (60) collected Self Defining memories (SDMs) using the self-defining questionnaire by Singer and Moffitt et al., 1991 (40), and carried out a multivariate analysis of variance to compare 25 people using alcohol and 28 controls. Those using alcohol recalled less specific SDMs compared to controls (C)(AD mean=1.60 (SD = 1.4); C mean=2.75 (SD = 1.9); t(51)=-2.49; p=0.016). People using alcohol reported significantly more negative valence (M=-0.26, SD = 1.4, *vs* M = 0.78, SD = 1.2, t(51)=-2.87; *p=0.006*), significantly more intense negative emotional responses (M = 2.9, SD = 3.1 *vs* M = 1.75, SD = 1.3; t51 = 3.09, *p=0.003*), and significantly lower positive emotional responses (M = 1.9, SD = 1.2 *vs* M = 3.0, SD = 1.4; t51=−2.83, *p=0.006*) in the SDMs recalled, compared to controls. Nandrino et al., 2014 (54) utilised the Autobiographical Memory Interview (AMI) (65) to assess episodic and semantic AM across three different lifetime periods including childhood (up to the end of high school), early adulthood (from 20 to 30 years of age), and recent adult life in 50 alcohol dependent individuals and 30 controls. Those consuming alcohol had significantly lower AMI scores for personal semantic (AD = 56.0(5.81); Control=59.7(5.16), t=2.84, *p=0.01*) and episodic (AD = 19(5.32); Control 24.34(2.54), t=6.02, *p=0.0001*) memory compared to controls. Episodic AM was significantly worse compared to controls in the childhood (AD = 6.08(1.98); Control=7.86(1.09); t=5.16); p=0.0001), early adulthood (AD = 6.44(2.05); Control=8.35(0.86); t=5.76; p=0.0001), and recent life subscales (AD = 6.46(2.18); Control=8.1(1.72); t=3.71; p=0.0001). Semantic memory was significantly worse compared to controls in the early adulthood and recent life subscales, but not in childhood. Nandrino et al., 2016 (55), similar to Nandrino et al., 2014 (54), utilised the AMI questionnaire (65) to investigate AM in 57 people using alcohol and 35 controls across the same three life periods. Those consuming alcohol, irrespective of abstinence period (short-term abstinence (STA) between 4 to 6 weeks or long-term abstinence (LTA) for at least 6 months), had significantly reduced episodic AM functioning (STA mean=20.28 (SD = 3.6); LTA mean=19.46 (SD = 4.42); C mean=24.2 (SD = 2.4), *p=0.000*) and semantic AM functioning (STA mean=55.65 (SD = 4.47); LTA mean=55.41 (SD = 4.6); C mean=58.62 (SD = 2.65); *p=0.008*). Using the Selective Reminding Test (SRT) (66), they reported that although episodic AM was significantly impaired in the 57 participants using alcohol, verbal episodic memory functions lacked statistical significance when compared to the 35 controls on the SRT (66) (STA mean=9.6 (SD = 2.8); LTA mean=9.85 (SD = 2.63); C mean=11.2 (SD = 3.25); *p<0.06*).

Nandrino et al., 2017 (31) assessed SDMs in terms of specificity, valence, integration of meaning, and theme in those consuming alcohol and 28 controls, additionally exploring the relationship between cognitive function and AM function. Those chronically using alcohol had significantly fewer specific SDMs (AD mean=55 (SD = 41); C mean=80 (SD = 57); *p<0.01*), and a significantly higher frequency of negative valence SDMs (AD mean=50 (SD = 37); C mean=30 (SD = 21); *p<0.01*) compared to the control group. The control group had a significantly higher frequency of the theme “achievement, mastery” recalled (*p<0.01*). The group who consumed alcohol also significantly recalled older memories compared to controls (t(259)=2.09, *p=0.04*, d=0.26). D’Argembeau et al., 2006 (51) utilised the AMT and MCQ tests to investigate AM recall and subjective experience, respectively, amongst the 20 who were drinking alcohol and 20 controls. Those using alcohol had significantly less frequent specific memories (t(38)=-3.5; *p=0.001*), but more frequent extended and categoric memories (t(38)= 2.04, p=0.048 and t(38)=2.35, *p=0.02*) compared to controls. De Groote et al., 2023 (61) found a significant increase in overgeneralised AMs in the group consuming alcohol compared to controls (AD mean=175 (SD = 72.02); C mean=158 (SD = 56.03); *p<0.001*). Self-statements were explored in relation to AM functioning, concluding that the 31 participants consuming alcohol reported significantly more negative self-statements in comparison to the 32 controls (*p=0.015*). De Groote et al., 2025 (62) agrees with these findings stating a significant increase in memories of negative valence in adulthood (AD mean=4.21 (SD = 2.61); C mean=3.19 (SD = 2.52); *p=0.023*) and within the past year (AD mean=3.54 (SD = 2.71); C mean=2.35 (SD = 2.02); *p=0.*009). Similarly to Nandrino et al., 2017 (31), AM recall regarding the theme of “achievement” was significantly lower in those consuming alcohol compared to controls (AD mean=22 (SD = 9.05); C mean=60 (SD = 21.28); *p<0.001*). Poncin et al., 2015 (52) assessed 61 participants who consumed alcohol (41 alcohol detoxifying (AD), 20 alcohol abstinent (AA) for ≥6 months) and 20 controls, using the AMT (42), to assess specific autobiographical recall and the Remember/Know procedure (69). Initially proposed by Tulving et al., 1985 (69), the Remember/Know procedure allows for the differentiation between the ability to remember an event as a conscious recollection as opposed to knowing an event due to feelings of familiarity. This is done so by using cued recall, free recall and recognition. The 41 alcohol detoxifying participants had significantly lower specific memories than their abstinent and control counterparts (AD-AA: t(77)=-2.86, *p<0.01*, η2 = 0.12, (-0.27; -0.02); AD-C: t(77)=-2.98, *p<0.01*, η2 = 0.13, (-0.27;-.03); AA-C: t(77)=-0.01, NS). These results suggest that specificity may recover with abstinence. Both AD and AA participants had a less pleasant perception of alcohol pictures compared to the control participants. Alcohol detoxifying participants had significantly fewer Remember and more Know responses when presented with memory- stimulating images compared to controls (Remember AD-AA: t(77)=-1.49, NS; Ad-C: t(77)=-4.07, *p<0.001*, η2 = 0.22, (-0.12;0.03); AA-C: t(77)=-2.06), NS; Know AD-AA: t(77)=1.3, NS; AD-C: t(77)=3.30, *p<0.01*, η2 = 0.16, (0.03;0.17); AA-C: t(57)=1.72, NS).

### Chronic cannabis use and autobiographical memory

The 2 papers assessing cannabis as the primary substance reported overgeneralised memories in those smoking cannabis compared to controls. Gandolphe et al., 2011 (56) assessed AM using the Williams and Scott AMT (42) in 69 people using psychoactive substances (51 cannabis, 18 polysubstance) and 38 controls. Those taking cannabis demonstrated significantly more general AMs compared to controls (p<0.001). Regarding the valence of the general AMs, the slopes were found to be homogenous, (F(4, 97) = 0.48; p=0.75), (F(4, 97) = 2.25, p = 0.07) for positive general memories and (F(4, 97) = 1.2; p= 0.32) for negative general memories. Pillersdorf et al., 2018 (53) used the Sentence Completion for Events from the Past Test (SCEPT) (43) and Fading Affect Bias (FAB) to assess the 47 participants using cannabis and 52 controls. Cannabis use led to increased overgeneralised memories with a mean difference of 0.12 (95% Cl 0.06-0.18). ANCOVA testing revealed a significant relationship between cannabis use and fading affect bias(F(1, 95) = 7.91, *p= 0.006*, ηp2 = 0.077). Affect fades at a slower rate for memories associated with unpleasant events in those using cannabis compared to controls, Mdiff = 1.61 (95% CI 0.67, 2.54), dunb = 0.69 (0.28, 1.09).

### Chronic opioid use and autobiographical memory

The 3 papers assessing opioids as the primary substance reported fewer specific memories in those using opioids compared to controls. Gandolphe et al., 2013b (57) utilised the AMT (42) in 73 people mainly using opioids (50 detoxifying, 23 under methadone maintenance) and 37 controls. Those consuming opioids, in both groups, had a significantly lower percentage of specific memories compared to controls (55.22(52–59 *vs* 73(67-79) *p<0.001).* These findings suggest that there is a lack of recoverability of specific AM retrieval. ANCOVA analysis revealed a statistically significant negative correlation between frequency of opioid consumption and the proportion of AMs classified as specific in nature (F(3,105)=-14.49, *p<0.001*). The influence of emotional awareness was also explored within this paper. Gandolphe et al., 2013a (58) conducted the AMT (42) in 30 people using opioid and 30 controls, which revealed fewer specific AMs in those taking opioids compared to controls (41.57% *vs* 64.58%). The ANCOVA also showed a significant effect of the type of consumption on the number of AMs recalled (F(1,57) = 7.30, *p<0.01*, partial n^2^ = 0.11). Gandolphe et al., 2013a (58) also explored the role of emotional avoidance strategy on specificity of AMs. Gandolphe et al., 2019 (59) assessed SDMs in terms of specificity, emotional valence and integration of meaning in 25 people who use opioids and 25 controls. Those consuming opioids had significantly fewer specific memories (F(1,48)=12.754; *p<0.001*; B=-1.295, 95% Cl 0.134-0.559), less integrated SDMs (F1,48 = 4.523, *p<0.05*, B=-0.833, CI(0.201; 0.940), OR=exp(-0.833)=0.435, Model -2LL=1104.992), and a much higher probability of recalling a neutral memory compared to controls (F1,48 = 6.885, *p<0.01*, B = 1.284, CI (0.318; 2.250), OR=exp(1.284)=3.611, Model -2LL=1208.894). The group using opioids recalled significantly more events with relationship themes compared to controls (F1,48 = 5.937, *p<0.01*, B = 0.722, CI (1.148; 3.691), OR = 2.059, Model -2LL=1088.505). The negativity or positivity of these events was not specified. The participants taking opioids also recalled significantly fewer events associated with the “achievement” theme (F1,46 = 4.699, *p<0.05*, B=-0.766, CI (0.232; 0.932), OR = 0.465, Model- 2LL = 1119.53).

### Associations between secondary outcome measures and autobiographical memory performance

#### Mood disorder

Throughout the studies, the influence of depression and anxiety on the relationship between substance abuse and AM retrieval was explored. A total of 8 papers found from the literature search explored the influence of depression on AM. Cuervo-Lombard et al., 2016 (60) found no association between depression and SDMs (ps>0.37) in those who use alcohol. D’Argembeau et al., 2006 (51) also reported no significant association (statistically significant p value adjusted to 0.0028 after correction for multiple covariates) between the Beck Depression Inventory (BDI) (70) score and percentages of specific (r=0.41, p=0.07) and categoric (r=0.44, p=0.06) memories in those consuming alcohol. Nandrino et al., 2017 (31) found a positive association between higher BDI-13 scores and higher negative memory recall. Gandolphe et al., 2013b (57) reported no significant association between the Hospital Anxiety and Depression Scale (HADS) score (71) or the level of depression and proportion of specific AM recall. In Gandolphe et al., 2019 (59), controlling for depression did not affect SDM emotional valence (F1,46 = 0.498, p=0.484; Model -2LL= 1247.206) in participants using opioids, or controls. Nandrino et al., 2016 (55) controlled for depression using ANCOVA as there was a significant difference between depression scores in those using alcohol compared to controls (STA = 12.85(10.1); LTA = 11.34(6.65); Controls=6.1(4.6); STA*vs*Controls p=0.001; LTA*vs*Controls=0.016). De Groote at al., 2025 (62) established greater HADS score in those who are alcohol dependent, compared to controls (Mean HADS Depression Score in AD = 5.92; Mean HADS Depression Score in C = 2.81; *p=0.*002), however this was not controlled. In the short term (between 4 and 6 weeks), there was a significant difference between recall on the SRT between abstinence and control participants (66), whilst in the long term (minimum of 6 months), there was no significant difference between abstinence and control participants. These results suggest that recoverability diminishes over time. Gandolphe et al., 2013a (58) reported a negative correlation between the level of memory specificity and depression (r=-.31, *p=0.01*). There was no significant difference in specific memory recall between the opioid-dependent group and the control group (Mann-Whitney U = 99.5, p=0.98). Cuervo-Lombard et al., 2016 (60) explored the influence of anxiety state on the characteristics of SDM but found no association (ps>0.12). However, an association between anxiety trait and specificity was identified (r=0.41, p=0.045) in participants consuming alcohol. D’Argembeau et al., 2006 (51) also reported no significant association between anxiety and AM in those who use alcohol (p>0.0028, with significance level adjusted to account for multiple comparisons). Nandrino et al., 2017 (31) found a positive association between anxiety levels and the frequency of negative memory recall. Gandolphe et al., 2013b (57) reported no significant association between the HADS score (71) and the proportion of specific AM recall in the controls or the dependent group (dependent individuals p=0.71; controls p=0.05; dependent and controls total p=0.05). In Gandolphe et al., 2019 (59), controlling for anxiety did not affect SDM emotional valence (F1,46 = 0.498, p=0.484; Model -2LL= 1247.206). Gandolphe et al., 2013a (58) reported no significant association between the level of memory specificity and anxiety in the opioid dependent individuals or the controls (p>0.05).

#### Severity and pattern of substance use

Cuervo-Lombard et al., 2016 (60) found no significant association between alcohol use severity and SDM. Poncin et al., 2015 (52) reported a significant negative association between the percentage of specific AM and consciousness of the severity of alcohol use (B=-0.36, *p<0.05*, (2.73;45.09), especially for specific memories not associated with alcohol experiences (B=- 0.41, R^2^ = 0.17, F(1,36)=7.22, *p<0.05*, (6.81;48.79)). There was no association between the score of consciousness of alcohol use and the Remember (F(1,36)=0.13) or Know (F(1,36)=0.44) responses (*p>0.05*). D’Argembeau et al., 2006 (51) found no significant association between the pattern of alcohol use, including number of previous detoxifications, days of alcohol abstinence, or daily amount consumed and AM (*p>0.05*). Nandrino et al., 2017 (31) also found no significant association between the length of alcohol abstinence and SDM (Pearson Correlation Coefficients: Level of Specificity=-0.20; Integration of Meaning=- 0.03; p>0.05). Gandolphe et al., 2019 (59) found earlier age of onset of opioid use was related to more neutral SDM recall. They found no significant association between length of abstinence, length of use, and age at onset with SDM specificity, valence, or integration of memories into life stories. De Groote et al., 2025 (62) appreciates the relationship between trauma memories and an increased severity of AUD, but this is not a component primarily explored in this study.

#### Fading affect bias

Fading affect bias is known to be closely related to emotional regulation. Pillersdorf et al., 2018 (53) explored this concept with relation to chronic substance use and AM impairment and concluded that there was no difference between those using cannabis and controls in affect fading for pleasant memories. Further follow-up multiple regression analyses were performed to assess the relationship between alcohol consumption and affect fading for unpleasant memories. At step 1, cannabis accounted for 4.84% of variance, and alcohol use for an additional 4.1% (*R2* = 0.11, *F*(1, 94) = 4.61, *p* = .034). At step 2, alcohol use was the sole significant predictor for affect fading bias for unpleasant memories. No direct (β = −0.06 (−0.13, 0.0008), *p* = .053) or indirect (β = −0.01 (−0.03, 0.01) effect was established when testing the role of overgeneral memory in the relationship between cannabis use and affect fading.

#### Theory of mind

Nandrino et al., 2014 (54) tested how Theory of Mind related to AM performance using the AMI (65), reporting an insignificant Theory of Mind function in participants using alcohol compared to controls (AD mean=19.7 (SD = 5.91); C mean=20.09 (SD = 7.76); p<0.82). Although both groups had an equal performance in the Versailles-Situational Intention Reading (V-SIR) (72), those using alcohol had fewer correct responses in the Reading the Mind in the Eyes Test (RMET) (73) compared to controls (22/36 *vs* 24/36). Pearson correlation revealed a significant positive association between the RMET scores and the timespan of alcohol use, and the semantic and episodic AMI scores (65) (alcohol consumption duration, R = 0.37, p < 0.001; AMI semantic, R = 0.27, p = 0.017; AMI episodic, R = 0.465, p < 0.001). Multiple regression analyses were subsequently performed, and the model using AMI scores (65) for both groups was statistically significant in the group where alcohol was used (F(2,49) = 6.062, p = 0.005) and in the control group (F(2,29) = 3.831, p = 0.035), and explained 20.5% and 23.5% of the variance of the RMET (73) performance, respectively.

### Publication bias and quality rating

All papers were assessed for risk of bias using the Joanna Briggs Institute Checklist for Analytical Cross-Sectional Studies (50). Reliable measures of exposure to substance, standard criteria to measure AM functioning, the addressing of confounding factors and adequate measure of outcomes were the only points of contention regarding bias assessment. Failure of 7 papers to state the qualifications and training of the examiners resulted in an unclear assessment of bias for the ‘adequate measure of outcomes’ domain. Regarding the ‘exposure to substance reliability measure’, all papers used one of the following to determine dependence: AUDIT (74), DSM-IV (75), DSM-V (76), Cannabis Use Disorder Identification Test-Revised (77) and Goodman’s Dependency Criteria (78). Gandolphe et al., 2011 (56) utilised the latter of these measures which lacked specificities compared to others. Poncin et al., 2015 (52) was the only paper not to address the confounding factor of depression, therefore introducing potential bias into the results. Gandolphe et al., 2011 (56), Gandolphe et al., 2013b (57) and Gandolphe et al., 2013a (58) did not specify the use of multiple examiners and therefore no deliberation regarding cognitive assessment is a potential source of bias in the ‘standard criteria to measure AM function’ domain. No papers were removed following bias assessment. The quality assessment results are outlined in **Table 2**.

## Discussion

This systematic review primarily aimed to explore the relationship between chronic psychoactive substance use and AM impairments, and secondarily to identify the association between secondary measures and AM performance. Results from the narrative synthesis showed a strong association between the chronic use of different substances (e.g. alcohol, cannabis, opioids) and AM impairments. For instance, those chronically using alcohol, cannabis, and opioids consistently retrieved less specific and more general AMs compared to controls. These results support a previous review conducted by Wright et al., 2022 (39), who found that participants with mental health, physical health, substance use, cognitive, and neurodevelopmental conditions had less specific and integrated memories, and more negative memories associated with their health condition or experienced trauma compared to controls. Albeit, their findings were limited to two studies conducted on participants consuming alcohol, and one study conducted on participants consuming opioids, and only utilised the Self Defining Questionnaire (SDQ) (40) as a measure of AM.

Progressively, this review considers findings from 13 papers, using a wide range of AM assessments, outlined in **Table 4** and **Table 5** in the Appendix. According to the capture and rumination (CaR), functional avoidance (FA), and executive control dysfunction (X) (CarFAX) model (22), overgeneralisation of AMs may result from an emotional avoidance strategy employed by individuals to avoid specific AMs charged with negative emotions. More specific evidence for the affect regulating function of functional avoidance is demonstrated by research showing that reduced specific AM retrieval is associated with the tendency to engage in avoidant coping styles (23). Functional avoidance may also be relevant for those chronically using substances as they suffer from poor emotional regulation (28, 31, 104). Indeed, Gandolphe et al., 2013a (58) reported a correlation between emotional avoidance strategies and reduced AM specificity in those chronically using opioids. However, this research did not test whether negative experiences would interact with functional avoidance to predict AM specificity in this population and was limited to one type of substance abuse. Executive dysfunction is another factor described in the CarFAX model that can impact AM retrieval (105). Executive dysfunctions refer to “deficits in executive resources that limit the successful retrieval of specific memories (e.g., by interfering with the ability to organise a retrieval search or to reject inappropriate responses)” (106). Notably, functional avoidance and executive dysfunctions are not mutually exclusive, and both mechanisms may be responsible for the overgeneralisation of AMs in people chronically using substances either alone or in interaction.

**Table 4:** Summary and explanation of each primary test performed in included studies.

Test	Component tested	Studies using this test	Explanation of test
AUTOBIOGRAPHICAL MEMORY TEST (42)	Autobiographical Memory Specificity	D’Argembeau et al., 2006 (51), Poncin et al., 2015 (52), Gandolphe et al., 2013b (57), Gandolphe et al., 2013a (58), Gandolphe et al., 2011 (56)	10 emotional cue words are presented and for each word, participants are asked to retrieve a specific event, lasting less than a day, and occurring at a particular place and time. Examples of what classify as specific and what do not are described to participants. A lack of response or a response that is not a memory is classified as an ‘omission’. Other responses are categorised into specific, extended and categoric memories (51).
AUTOBIOGRAPHICAL MEMORY INTERVIEW (65)	Explores the episodic and semantic components of autobiographical memory in different stages of life	Nandrino et al., 2014 (54), Nandrino et al., 2016 (55)	40 questions assessing semantic AM (maximum score 21 per life period) and nine questions assessing episodic AM (maximum score 9 per life period). These are measured for childhood, early adult life (20–30 years old) and recent life. These scores are then summed together to provide an overall semantic AM and an overall episodic AM score, the ranges are as follows: · Normal 19-21 · Borderline 17-19 · Pathological <17 Points system is as follows: · Specific events recalled = 2 points · Vague personal memory = 1 point · No response/No memory = 0 points (54, 65)
SENTENCE COMPLETION FOR PAST EVENTS TEST (43)	Autobiographical Memory Specificity	Pillersdorf et al., 2018 (53)	11 neutral sentence starters are provided, and the participants must complete the sentence with a memory. Participants then had to classify the memory themselves in terms of specificity and this was also assessed by an examiner (43, 51)
SELF-DEFINING QUESTIONNAIRE (40)	Self-Defining memory specificity	Cuervo-Lombard et al., 2016 (60), Nandrino et al., 2017 (31), Gandolphe et al., 2019 (59)	A self-defining memory is described to individuals and then they are asked to describe five SDMs, including specific details about the event and time of occurrence as well as a summary of the event and memories are then assessed according to themes present, associated valence and specificity (40, 60)
SELECTIVE REMINDING TEST (79)	Verbal Episodic Memory Function	Nandrino et al., 2016 (55)	15 words are read at a pace of 1 word per 2 seconds to the participants, who are then asked to repeat the list of words back as quickly as possible. If any words are forgotten, the process is repeated until all words are recalled (maximum ten times) The score given is the number of correct words recalled. 20 minutes later, the participant is asked to list off the items without hearing the words again (55, 79).
I AM MEMORY TASK (80)	Autobiographical Memory Specificity	De Groote et al., 2023 (61)	Participants must list 3–10 statements of how they view themselves, “create a list of adjectives, personal values or traits which characterise you best”. They then must recall 8 autobiographical memories which best illustrate these statements. Participants must then date each memory recalled to obtain the temporal distance of the retrieved memory (80).
COGNITIVE AVOIDANCE QUESTIONNAIRE Sexton et al., 2008 (81)	Thought suppression, though substitution, distraction, avoidance	Gandolphe et al., 2013a (58)	Questionnaire consisting of 25 items which have the potential to trigger intrusive thoughts and therefore employ cognitive avoidance strategies. Participants are given scenarios which fit in to the following: 1. Thought Suppression 2. Thought Substitution 3. Distraction 4. Avoidance of Threatening Stimuli 5. Transformation of Images into Thoughts Statements describing the use of the above strategies are ranked by participants from 1=not at all typical, to 5=extremely typical. The higher the overall score, the greater the tendency to cognitively avoid threatening stimuli (58, 81).
CHILDHOOD TRAUMA QUESTIONNAIRE (82)	Presence of traumatic memories	Nandrino et al., 2016 (55)	28 item questionnaire which assess childhood trauma from:
			1. Emotional abuse 2. Physical abuse 3. Sexual abuse 4. Emotional neglect 5. Physical neglect
			Responses to each item are answered from 1=never true, to 5=very often true. The higher the score, the more severe the exposure to childhood trauma. A score is calculated for each of the five sections above (55, 82).
SHORT-FORM MEMORY EXPERIENCES QUESTIONNAIRE (64)	Subjective experience of AMs	De Groote et al., 2025 (62)	63-item self-report scale measuring 10 phenomenological qualities of AMs:
			• Vividness • Coherence • Accessibility • Time Perspective Sensory Details • Visual Perspective • Emotional Intensity • Sharing • Distancing • Valence
			Each memory is rated on the subjective experience of said memory, several other scales are also used to measure the psychological distress associated with the memory. – need to reference!

SDQ, Self-Defining Questionnaire; AMT, Autobiographical Memory Test; SCEPT, Sentence Completion for Events from the Past Test; AMI, Autobiographical Memory Interview; SRT, Selective Reminding Test; AM, Autobiographical Memory; N, Number; M, Mean; SD, Standard Deviation; n/a, Not Available; SDM, Self Defining Memories; OGM, Overgeneralised Memories; FAB, Fading Affect Bias; AD, Alcohol Dependent; IAMT, I Am Task; MEQ-SF, Short Form Memory Experiences Questionnaire.

**Table 5:** Facets of AM.

Main domain	Subtypes	Other names	Definition	Tests	Tests used in this review
Autobiographical Memory	Episodic Autobiographical Memory	Personal Episodic Memory, Self-Centred Memory, Memory for Life Events, Childhood Memory, Narrative Memory	An individual’s memory for events or experiences that occurred in their own life (83).	Autobiographical Memory Interview (65) Autobiographical Fluency Task (84) I Am Memory Task (85) Autobiographical Recollection Test (86) Life Chapters Task (66) Early Memories Test (87)	Autobiographical Memory Interview (65) I Am Memory Task (85)
	Semantic Autobiographical Memory	Personal Semantic Memory, Self-Centred Memory, Memory for Life Events, Childhood Memory, Narrative Memory	An individual’s memory for personal factual knowledge and concepts (83).	Autobiographical Memory Interview (65) Autobiographical Fluency Task (84) Selective Reminding Test (79)	Autobiographical Memory Interview (65) Selective Reminding Test (79)
	Trauma Memories	Negative Life Events	Memories that are formed after an experience triggering high levels of negative emotional arousal (88).	Autobiographical Memory Questionnaire (89) Childhood Trauma Questionnaire (82)	Childhood Trauma Questionnaire (82)
				Life Events Checklist – 5 Memory Characteristics Questionnaire (90)	
				Impact of Event Scale – Revised (67)	
				Centrality of Event Scale (91)	
				Life Stressor Checklist – Revised (92)	
				Potential Stressful Events Interview (93)	
				Stressful Life Events Screening Questionnaire (94)	
				Trauma History Questionnaire (95)	
	Voluntary Autobiographical Memory	Intentional Remembering of Personal Memory	Controlled and strategic approach is required to recall a personal memory (96).	n/a	n/a
	Involuntary Autobiographical Memory	Unintentional Remembering of Personal Memory	No preceding attempt is required to recall a personal memory (95).	Impact of Event Scale – Revised (67)	n/a
				Responses to Intrusions Questionnaire (97)	
				Involuntary Autobiographical Memory Inventory (98)	
	Autobiographical Memory Specificity	Overgeneralised Autobiographical Memory	Refers to the retrieval of a specific personal memory lasting a day or less, recall of a highly contextualised event (99).	Autobiographical Memory Test (42) Sentence Completion for Events from the Past Test (43)	Autobiographical Memory Test (42) Sentence Completion for Events from the Past Test (43)
				Galton-Crovitz Test (100)	
				Autobiographical Recollection Test (86)	
	Self-Defining Memory	Central Life Events	Memory of personal events which are highly relevant to the identity of an individual (101).	Self-Defining Memory Task (102)	Self-Defining Memory Task (102)
				Twenty Statements Test (103)	I Am Memory Task (80)
				I Am Memory Task (80) Centrality of Event Scale (91)	Short-Form Memory Experiences Questionnaire (64)
				Short-Form Memory Experiences Questionnaire (64)	

Evidence for the role of executive functions in AM impairments suggests that chronic substance use may not only cause structural and functional impairments in brain regions in charge of AM formation and retrieval such as the hippocampus (107), but also changes in prefrontal cortices in charge of executive functions such as the medial prefrontal cortex (mPFC) (37). Neuroimaging evidence has pointed out that semantic AM and episodic AM impairments are related to structural impairments in the mPFC (108). For instance, semantic AM impairments are related to structural impairments in the left mPFC and medial temporal lobe, whereas impairments in the right mPFC and medial temporal lobe are associated with episodic AM impairments (108). According to a recent meta-analysis conducted by Zeng et al., 2024 (109), chronic substance use is associated with lower resting-state functional brain activity and reduced grey matter volume (GMV) in the bilateral mPFC among other brain regions (e.g. ACC). Therefore, considering that the current review underlined diminished episodic and semantic AM performance in people who chronically use substances compared to controls (31, 55), it could be postulated that AM impairments may be related to executive dysfunctions caused by the daily consumption of psychoactive substances. Albeit, direct causality cannot be inferred due to the cross-sectional design of the studies pooled for the current review, the study conducted by Gandolphe et al., 2011 (56) reported that the group of participants with a diagnosis of cannabis dependence and the group of participants with a diagnosis of poly-substance dependence recalled more overgeneralised AMs compared to those who regularly use cannabis, those who occasionally-regularly use cannabis, and healthy controls. This finding might imply a dose-effect relationship as individuals with a higher frequency of substance use may suffer more severe AM impairments compared to individuals with a lower frequency of substance use. The impact of dose level needs to be the focus of future work.

### Relationship between emotions and AM impairments

In papers primarily focused on the chronic consumption of alcohol or cannabis, a significant increase in overgeneralisation of AMs is observed across all studies, with a lack of consensus regarding the valence of retrieved AMs. Pillersdorf et al., 2018 (53) supports previous literature by Troup et al., 2016 (110), suggesting that cannabis use increases the recognisability of negative emotions compared to positive emotions, which should decrease the frequency of positive valence AMs. Previous literature by Cousijn et al., 2012 (41) concluded an increased grey matter volume in those who heavily use cannabis. As the grey matter holds accountability for the processing of emotions (Chiao CC, Lin CI, Lee MJ), these structural neurological changes could potentially account for the overgeneralisation of AM. Mitchell et al., 2015 (111) suggests an increase in accessibility of positive valence AMs with alcohol consumption, accounted for by the euphoric sensation induced by endorphin release from the nucleus accumbens. Whereas a decrease in negative valence AMs can be explained by dopamine depletion resulting from alcohol consumption causing a negative interpretation of positive memories (112). However, this is contradicted by findings from subsequent studies (31, 60–62), which all suggest an increase in frequency of negative valence memories and a decrease in frequency of positive valence memories. Understanding the bidirectional relationship between negative emotions and alcohol consumption is essential to determine the role of the Mood Congruent (26) and Mood Dependent Effects (27) in the consolidation and retrieval of AMs. If a negative mood drives alcohol consumption then the Mood Dependent Effect (27) and Mood Congruent Effect (26) can be used to explain the observed increase in negative valence memories in alcohol dependent individuals. The same principle can be applied to explain the impaired ability for alcohol dependent individuals to recall AMs of positive valence.

The inconsistency in controlling for depression may account for the statistically insignificant change of negative valence memory frequency observed by D’Argembeau et al., 2006 (51). Corcoran et al., 2003 (113) support the findings regarding reduced Theory of Mind functioning in individuals consuming alcohol being responsible for the respectively reduced AM function as a lack of comprehension of surrounding emotions reducing capability to recall specific AMs according to the Mood Congruent and Mood Dependent effects (26, 27). Contrastingly, the frequency of positive valence AMs in people using opioids was decreased. Adams et al., 2003 (114) and Lang et al., 1995 (115) categorise emotion into avoidance-orientated, sadness and fear, and approach-orientated, pleasure and anger, suggesting heightened approach-orientated emotions with opioid consumption, and respective inhibition of avoidance-orientated emotions. If anger prevails over pleasure, positive valence decreases, according to the Mood Congruent Effect (26), which proposes an increase in valenced memories that are consistent with mood. In Gandolphe et al., 2019 (59) a significant increase in neutral memories is observed with chronic opioid consumption and can be explained by the interaction of trauma and emotions.

Gandolphe et al., 2013b (57) is the only study to utilise the Cognitive Avoidance Questionnaire (CAQ) (81), as shown in **Table 4**, and in doing so demonstrates significantly higher scores on the CAQ in opioid consumers compared to controls. De Groote et al., 2025 (62) concurs with these findings, stating an increase in distancing and a decrease in sharing of AMs in all stages of life, excluding childhood for which the opposite was true. This suggests a loss of identity when comparing the person they are today with who they once were; individuals with alcohol use disorder do not experience a sense of self-continuity. Finally, no consistent effects were found for the impact of depression and anxiety on the relationship between substance abuse and AM with many studies showing no impact of these mood variables.

### Strengths and limitations

This systematic review presents several strengths. An extensive, multilingual database search was carried out with a stringent screening and inclusion process. Efforts were made to contact authors of the studies where data was incomplete or unclear. Publication bias was adequately assessed using the JBI Checklist (50), and the 13 papers included were deemed to be of good quality overall. Data is presented from studying the relationship between psychoactive substance use, mainly alcohol, cannabis, and opioid, and AM impairment. Data was analysed from four countries within the past 12 years. Those who use substances were selected using the DSM IV or V criteria across the studies. Participants were of a wide age range, and the group using substances *vs* control group sizes were similar in some of the studies (31, 51, 53, 58, 60, 62). This is a strength as it encompasses a greater proportion of the population, making findings more applicable to a greater number, yet contrastingly makes findings less specific.

There is evidence that the impact of age depends on the measure of autobiographical memory recall employed. Mair et al. (2021) (111) found that the cueing paradigm resulted in larger ABM specificity deficits in older as opposed to younger adults. Thus, the extent of autobiographical deficits reported in the current narrative synthesis may be impacted by age and assessment method. In most studies, substance use and control groups were matched for age (58) and education (31, 51, 52, 54, 55, 57, 60, 62). A range of tests were used across the studies to measure the primary outcome, and the procedures were well explained and replicable. Some studies also reported measures to reduce bias and inter-rater variability such as independent rating of test scores by multiple raters (31, 51, 53–55, 60).

Many studies also explored secondary outcome measures in relation to AM performance, including but not limited to depression, anxiety, severity and pattern of dependence, cognitive function, emotional awareness, Theory of Mind, and fading affect bias. This allows for holistic analysis of the relationship between chronic psychoactive substance use and AM impairment by accounting for numerous influential factors beyond the direct influence of substance use itself.

The review also presents several limitations that pave the way for future research. The generalisability of findings is limited demographically as data is presented from France, Switzerland, Belgium, and Canada, on participants of unknown ethnic background, only one paper provides a breakdown of participant ethnicities (53). There was a significantly higher number of male participants than female participants across all studies except for one study (53) where the opposite was true. The sample sizes across all 13 studies are small, with the largest cohort including 73 people who use substances and 23 controls (57).

Across the studies, the length of substance use and/or abstinence at the time of assessment was highly variable amongst the participants. This is good for data collection at various time points but also poses a challenge to drawing definitive conclusions. Of the three papers exploring AM recovery post-abstinence, only those who were chronically using alcohol were studied, and cross-sectional designs were employed. Additionally, the length of abstinence period was not consistent between studies.

A further limitation identified is the inconsistency in AM screening tools between the 13 papers – a total of 9 tools were used, with each assessing AM from a slightly different approach which naturally provides potential discretion in interpretating and comparing these results. Finally, a lack of standardization for the methods used to assess autobiographical memory could also impact results. For example, the AMT was used in multiple studies. A review by Griffith et al., 2012 (116) has highlighted that variability in the administration of the AMT in oral versus written format, the type of cue words employed including whether they are concern related, the application of time-limits and scoring procedures can impact results and their interpretation.

### Areas for future research

Three papers in this review (52, 55, 57)) explored the important question of whether AM can be recovered following a period of abstinence, with De Groote et al., 2025 (62) being the only paper to relate findings to risk of future relapse. Further findings from these studies are not consistent. A previous meta-analysis, conducted by Stavro et al., 2013 (117), concluded that neurocognitive function partially recovers after an alcohol abstinence period of more than one year. However, Schulte et al., 2014 (118) identified several mediating factors to this relationship including family history of alcohol dependence, number of detoxifications, and heavy smoking in addition to the duration of abstinence. There is a need for longitudinal data collection to shed light on the temporality of findings and account for identified and potential confounding variables. Thus, future studies should use a design with assessments of autobiographical memory over treatment course, including the recovery period accompanied by using rigorous structured interviews/measures to assess age, duration of abstinence, family history of alcohol dependence and other confounding factors like depression and trauma. To improve standardization, measures of autobiographical memory with robust evidence for their validity and reliability, like the autobiographical memory interview (Koppelman.et al., 1989 (65)), should be employed. When the AMT is used, there should be a careful description of the methodology and scoring criteria. Using AMT instructions that have been employed in previous addiction research would facilitate comparison of findings. Finally using assessment tools like the Addiction Severity Index (119) which have strong evidence for reliability and validity would standardize the assessment of addiction history and key demographic information.

Gender differences are another area for future research. The gender imbalance identified in the current studies with the focus on males is consistent with a wider concern that addiction research has traditionally focused on males (Tuchman 2010) (120). Our findings on autobiographical memory confirm this bias. There are important reasons to focus on gender in future research. Females as opposed males who engage in substance abuse have higher psychiatric co-morbidities like depression and trauma (Zilberman et al., 2003) (79). These factors are associated with difficulties in retrieving autobiographical memory (18, 19), and could be important factors that contribute to autobiographical memory impairments in women who are substance abusers. Further improved autobiographical memory retrieval could reduce the risk for substance abusee relapse by reducing vulnerability for depression and trauma (18).

Executive function impairments occur with many forms of substance abuse (116) while also playing a key role in AM retrieval. Investigating executive function in relation to autobiographical memory retrieval could provide more insight into the processes involved in memory retrieval. Guler et al. (2019) (120) found the executive functions of inhibitory control and flexibility were associated with greater specific retrieval in neurotypical individuals. However, the type of cue had no effect. Thus, focusing on how specific executive functions are associated with AM retrieval in substance abuse is important for future work.

It is important to have more research on mechanisms so that interventions for improving AM function can be developed for substance abuse. There were no papers in the review that focused on interventions for improving AM functioning in this group, despite evidence that Memory Specificity Training (MeST) results in an increased ability to retrieve specific AMS in depression (19). Finally, the implications of AM retrieval deficits for psychological well-being through associations with social problem-solving, social interactions and identity have not been explored in those who use substances. Poor functioning in these domains increases the risk of developing or relapsing in substance abuse disorders. AM retrieval plays a key role in these functions (119), highlighting that interventions for improving Am retrieval in substance abuse populations may have important treatment implications.

## Conclusion

This novel systematic review concludes that chronic consumption of psychoactive substance use, including cannabis, alcohol, and opioids, is associated with specific impairments in AM. The associations between AM performance and secondary outcome measures including mental health, severity and pattern of substance use, cognitive and emotional functions, Theory of Mind and Fading Affect Bias were explored. Areas for future research were also outlined to ultimately improve the clinical management of this population.

## Data Availability

The original contributions presented in the study are included in the article/supplementary material. Further inquiries can be directed to the corresponding author.
